# Intrathecal baclofen in paroxysmal sympathetic hyperactivity: Impact on oral treatment

**DOI:** 10.1002/brb3.1124

**Published:** 2018-09-28

**Authors:** Elke Pucks‐Faes, Gabriel Hitzenberger, Heinrich Matzak, Giulio Verrienti, Robert Schauer, Leopold Saltuari

**Affiliations:** ^1^ Department of Neurology Hochzirl Hospital Zirl Austria; ^2^ Research Unit for Neurorehabilitation South Tyrol Bolzano Italy

**Keywords:** autonomic nervous system diseases, baclofen, brain injuries, sympathetic nervous system, traumatic

## Abstract

**Introduction:**

Intrathecal baclofen (ITB) is a commonly used treatment in severe spasticity. The main objective of this study was to assess the impact of ITB on reduction or withdrawal of oral drugs in patients with paroxysmal sympathetic hyperactivity (PSH) after severe traumatic brain injury.

**Methods:**

We retrospectively evaluated 20 patients with PSH due to severe traumatic brain injury who were treated with ITB in a primary care and referral center of neurology. Rates of and time until complete withdrawal or possible reduction in oral baclofen and oral propranolol after ITB treatment were calculated. Moreover, vegetative parameters (heart rate and blood pressure) as well as hypertonic attacks were assessed.

**Results:**

The median time of complete oral baclofen disposal was 5 ± 3.7 (CI 95% [2.9–7.1], range 0–14) days after ITB pump implantation in 20 of 20 patients, and the median time of complete oral propranolol disposal was 24 ± 62.97 (CI 95% [−7.87–55.87], range 5–191) in 15 of 20 patients. With ITB treatment, PSH improved promptly in all patients with alleviation of heart rate and blood pressure as well as reduction in attacks with motor phenomena. ITB treatment was safe and without complications.

**Conclusions:**

ITB might facilitate cutting back or dispensing with other conventional oral drugs, such as oral baclofen and oral propranolol. Our study provides further evidence that ITB treatment should be considered in patients with otherwise medically refractory PSH in severe traumatic brain injury. Further prospective multicenter studies are needed to confirm the findings of this study.

## 1 INTRODUCTION

The course of severe brain injury is often complicated by excessive autonomic dysfunction with reported incidences varying between 8% and 33% with higher incidences in more severe cases (Baguley et al., 1999; Becker, Sure, Petermeyer, & Bertalanffy, 1999; Dolce et al., 2008; Fernández‐Ortega et al., 2006; Hendricks, Heeren, & Vos, 2010; Jennett et al., 1977; Rabinstein, 2007). The most common term for this major complication cited in the literature is *dysautonomia*. In a review on acquired brain injury, the more specific and clinically relevant term “paroxysmal sympathetic hyperactivity” (PSH) was proposed as this might better reflect the consistency of sympathetic features (Perkes, Baguley, Nott, & Menon, 2010). PSH was defined as episodes of autonomic instability with paroxysmal increases in at least five features of sympathetic hyperactivity and motor features (increases in heart rate, respiratory rate, blood pressure, temperature, sweating, pupillary dilation, decerebrate posturing, decorticate posturing, spasticity, hypertonia and/or dystonia, teeth grinding, and agitation; Baguley et al., 1999; Perkes et al., 2010). In 2014, a consensus statement of an international expert group on conceptual definition, nomenclature, and diagnostic criteria of PSH was published utilizing a Delphi approach.

An assessment measure was developed combining the diagnostic likelihood of PSH and severity of clinical features (Baguley et al., 2014).

The etiology of brain injury was suggested to be a major and non‐modifiable determining factor for the course of PSH, the response to intrathecal baclofen (ITB), and the long‐term outcome. Compared to hypoxic brain injuries, patients with traumatic brain injuries needed lower doses of baclofen, showed a significant improvement of PSH, and had a higher level of consciousness recovery and better functional abilities in the long term (Hoarau, Richer, Dehail, & Cuny, 2012). Additionally, early recognition and treatment of PSH were suggested to play a crucial role in minimizing secondary brain injury due to metabolic changes as well as posturing being associated with increased noradrenaline concentrations which might independently predict poor outcome after traumatic brain injury (Hamill, Woolf, McDonald, Lee, & Kelly, 1987; Hoarau et al., 2012).

Conventional treatment with oral therapy including sedative–analgesic drugs, muscle relaxants, antiadrenergic drugs, and oral baclofen often fails to control PSH sufficiently. Moreover, continuous sedation and analgesic drugs might prolong respiratory support in the intensive care unit and thus delay early neurorehabilitation and recovery, compromise physiotherapeutic activity, and possibly worsen functional outcome.

ITB has successfully been used in severe spinal as well as in severe supraspinal spasticity for more than 30 years where adequate response to oral antispastic medication has not been observed (Albright, Barron, Fasick, Polinko, & Janosky, 1993; Albright, Cervi, & Singletary, 1991; Armstrong et al., 1997; Becker, Alberti, & Bauer, 1997; Coffey et al., 1993; Dario, Stefano, Grossi, Casagrande, & Bono, 2002; Meythaler, DeVivo, & Hadley, 1996; Meythaler, Guin‐Renfroe, Grabb, & Hadley, 1999; Meythaler, McCary, & Hadley, 1997; Penn & Kroin, 1985; Penn et al., 1989; Rawicki, 1999; Rifici et al., 1994). The use of oral baclofen is often limited by poor tolerability, especially in higher dosages in severe cases of spasticity. Moreover, sufficient concentrations in the central nervous system can only be achieved by intrathecal application of baclofen (Knutsson, Londblom, & Mårtensson, 1974). A switch to ITB is also indicated as intolerable side effects to oral baclofen often occur.

Successful treatment of PSH with ITB has been demonstrated in numerous studies in the literature, albeit mainly in small patient series (Becker et al., 1999; Becker, Benes, Sure, Hellwig, & Bertalanffy, 2000; Cuny, Richer, & Castel, 2001; François et al., 2001; Turner, 2003). The largest study on patients with severe traumatic brain injury and dysautonomia treated with ITB therapy comprises 43 patients (Hoarau, Richer, Dehail, & Cuny, 2012). The authors reported the long‐term disorders of consciousness after a mean follow‐up of 10 years. A low level of consciousness recovery and early development of severe and persistent dysautonomic symptoms were associated with a poor long‐term outcome.

However, treatment of PSH with ITB has not yet been established as information on effectiveness after treatment failure of sedative–analgesic drugs, antiadrenergic drugs, and oral baclofen is limited. Moreover, there is no information on efficacy concerning impact of ITB treatment on co‐medication in terms of potentially making other drugs in the management of PSH dispensable. The main aim of this study was to assess the effect of ITB in facilitating withdrawal of other oral drugs applied before ITB therapy to treat PSH. Furthermore, we report on our experience with ITB to control PSH in patients with severe traumatic brain injury.

## 2 MATERIALS AND METHODS

### 2.1 Subjects and study design

We retrospectively evaluated 78 patients with a severe traumatic brain injury, who were treated with ITB in the Department of Neurology, Hochzirl Hospital, Zirl, Austria, between 1997 and 2015. Inclusion criteria were (a) PSH refractory to maximum doses of conventional non‐invasive treatments, such as sedative–analgesic drugs, muscle relaxants, antiadrenergic medication, and oral baclofen; (b) ITB therapy was applied to control PSH. Included patients were either referred to our hospital from an intensive care unit of the hospital catchment area or from a district hospital for a second opinion and evaluation. Insufficient patient data were an exclusion criterion.

### 2.2 Data collection

Collected data included demographic and injury details, for example, the initial Glasgow Coma Scale (Teasdale & Jennett, 1974), information on clinical details and course, as well as rehabilitation outcome. Regarding medical treatment, the oral therapeutic schemas of conventional medication, applied before ITB device implantation to treat PSH, were compared to the medication schemas at discharge from hospital. Oral baclofen and oral propranolol were further analyzed regarding time and extent of possible reduction or complete withdrawal after implantation of the ITB pump. The time intervals between the initiating event and first ITB administration via an external pump were calculated. Furthermore, data on maximal dosage of ITB during the evaluation phase with the external pump, as well as on dosage of ITB at discharge from hospital and on last follow‐up, were collected.

The effect of ITB on PSH was evaluated comparing vegetative parameters (heart rate and blood pressure) as well as attacks with muscle hypertonia before and after ITB pump implantation. An assessment was made from physicians’ and nurses’ documentation in the patient files according to the regular monitoring of vital signs (heart rate and blood pressure) as well as description of the clinical presentation (attacks with motor phenomena).

The median percentage of days with episodes of a heart rate ≥100 beats/min was assessed between the period from admission to our hospital until the time of ITB pump implantation and compared to the period from readmission after ITB pump implantation until discharge from hospital. Likewise, the median percentage of the daily number of episodes with systolic blood pressures >160 mm Hg was assessed between admission to our hospital until implantation of the ITB pump implantation and compared to the period of readmission after ITB pump implantation until discharge from hospital. In addition, the mean and maximum heart rates as well as the mean and maximum systolic blood pressure levels were compared over a period of one week before implantation of the ITB pump and during one week before discharge from hospital (heart rate and blood pressure measured at least three times a day). Number of attacks per day with motor phenomena comprising decerebrate or decorticate posturing, hypertonia, and/or dystonia, teeth grinding and agitation were counted for the period of one week before ITB pump implantation and compared to a period of one week before discharge using an arbitrary scale from 0 to 2 points; three or more episodes per day (0 point), one or two episodes per day (1 point), and zero episodes per day (2 points).

Evaluation of functional outcome was measured by assessing the remission phase according to the classification of Gerstenbrand at time of admission and discharge from hospital (Das, 1967). The “Innsbruck apallic syndrome remission scale” comprises eight phases describing possible recover phases from full stage. The Ashworth Scale to grade the impact of ITB on spasticity was compared prior to (at time of admission) and after applying ITB treatment (at time of discharge from hospital). Furthermore, the Glasgow Outcome Scale was used to evaluate the patients’ functional outcome at discharge (Jennett & Bond, 1975).

### 2.3 Intrathecal baclofen treatment

Intrathecal baclofen was first administered as a continuous application via lumbar route through an intrathecal catheter after subcutaneous tunneling. Subsequently, the intrathecal catheter was connected with an external programmable pump enabling continuous as well as bolus mode of baclofen application. The duration of test period varied among patients on an individual basis depending on the time when a positive response as defined below occurred. The decision to remove the intrathecal catheter and proceed with the implantation of an ITB pump was made in an interdisciplinary setting (attending physicians, nurses, and therapists) with video documentation of the clinical situation before and after start of ITB treatment. Single‐shot antibiotic medication administered to all patients on the day of intrathecal catheter application avoids any possible infectious side effects resulting from the procedure.

According to an earlier study, a positive response to ITB was defined as an alleviation of dysautonomia symptoms within 24 hr, recurrence of symptoms within 24 hr after stopping the ITB evaluation, and a 2‐point decrease in muscle tone (Ashworth Scale) in the upper or lower limbs (Turner, 2003). After a positive response, the intrathecal catheter was removed and the patient transferred to the Department of Neurosurgery, where the catheter was surgically replaced with subcutaneous implantation of a continuous infusion pump in an abdominal site delivering baclofen (Medtronic Synchromed II^®^). Occurring side effects of ITB were assessed.

### 2.4 Statistical analysis

Qualitative and quantitative variables were summarized using descriptive statistics.

For testing statistical significance of the outcomes before and after ITB therapy, either one‐tailed Student's *t* tests for paired samples or Wilcoxon signed‐rank tests were used, with a significance level of 0.05. In the first case, the normality assumption was assessed visually as well as by applying a Kolmogorov–Smirnov test with a significance level of 0.05. As the measurements of sweating episodes and hypertonic attacks were not normally distributed, a Wilcoxon signed‐rank test was used instead of a paired *t* test. Wilcoxon signed‐rank tests were also used to test for significant differences between the ordinal outcomes before and after ITB therapy.

According to the Austrian law on retrospective research, this study did not require the approval of the ethics committee.

## 3 RESULTS

Twenty patients (16 men, mean age 28 ± 11 years, range 8–52 years) with severe traumatic brain injury and ITB treatment for PSH entered the final analysis. Six of 20 patients were referred from an intensive care unit of the hospital catchment area and 14 of 20 patients from a district hospital for a second opinion and evaluation. In all of them, PSH was refractory to maximum doses of conventional non‐invasive treatments. In the remaining 58 of 78 patients, ITB was applied to control the severe spasticity, while occurring PSH could be controlled with conventional non‐invasive therapies sufficiently. Demographic and clinical details including treatment as well as outcome parameters of the 20 patients are summarized in Table 1. The median Glasgow Coma Scale of severe traumatic brain injury was 4 ± 2.3 (CI 95% (Becker et al., 1999; Dolce et al., 2008; Rabinstein, 2007) range 3–12).

**Table 1:** Demographic data, clinical details, treatment and outcome parameters of the 20 patients with severe traumatic brain injury and intrathecal baclofen treatment for paroxysmal sympathetic hyperactivity

No	Sex	Age at accident (years)	GCS	Clinical features of paroxysmal sympathetic hyperactivity	Time event to external pump (days)	Medication before pump implantation	Medication at discharge	Mean heart rate before/after^a^ (bpm)	Mean SBP before/after^a^ (mmHg)	Mean score for hypertonic attacks per day before/after^a^	GOS	Remission phase at admission	Remission phase at discharge	Ashworth Scale at admission	Ashworth Scale at discharge	Follow‐up (months)
1	m	25	6	Decorticate posturing, flush, sweating, teeth grinding, bulldog phenomenon, not obeying any commands	79	Baclofen 100 mg/d Propanolol 60 mg/d Morphine 20 mg/d	ITB 180 µg/d	114/82	133/123	0/2	2	1	4	4	2	12
2	m	42	4	Decorticate posturing, lateroflexion of head, sweating	302	Baclofen 100 mg/d Propanolol 120 mg/d	ITB 260 µg/d Propanolol 20 mg/d	86/104	122/120	0.14/1.71	3	2.5	2	3.5	2	5
3	m	23	3	Decorticate posturing, teeth grinding lateroflexion of head	30	Baclofen 100 mg/d Clonazepam 1 mg/d Diazepam 10 mg/d Fentanyl 50 µg/hr	ITB 200 µg/d Clonazepam 1 mg/d	96/87	118/112	0/1.14	3	1	1	4	2.5	32
4	m	19	4	Deocorticate/decerebrate posturing, bulldog phenomenon	122	Baclofen 100 mg/d Propanolol 120 mg/d	ITB 370 µg/d Propanolol 40 mg/d	95/79	130/126	0/1.71	2	1	2.5	4	2.5	90
5	m	35	7	Sweating, teeth grinding, decorticate posturing, bulldog phenomenon	43	Baclofen 100 mg/d Tizanidin 8 mg/d Buprenorphine 52.5 µg/hr	ITB 200 µg/d	94/85	129/115	0/1.71	3	1	3	3.5	2.5	73
6	f	8	8	Decorticate posturing, opisthotonus	559	Baclofen 75 mg/d Propanolol 10 mg/d	ITB 560 µg/d	92/98	102/122	0.43/1.86	3	1	3	4	2	28
7	m	25	12	Teeth grinding, bulldog phenomenon, sweating, opisthotonus, decerebrate posturing	141	Baclofen 100 mg/d Propanolol 20 mg/d Lorazepam 1 mg/d Hydromorphon 6 mg/d Phenobarbital 300 mg/d	ITB 160 µg/d Lorazepam 1 mg/d Clonazepam 1.5 mg/d Hydromorphon 2 mg/d	92/75	119/103	0/1.43	2	1	1	4	2	31
8	m	41	5	Decorticate posturing, sweating, bulldog phenomenon	81	Baclofen 100 mg/d Propanolol 80 mg/d Tizanidin 16 mg/d	ITB 145 µg/d	102/80	123/107	0.29/1.43	2	1	1	3	1	76
9	m	24	5	Decorticate posturing, opisthotonus lateroflexion of head, loss of pursuit eye movements	148	Baclofen 125 mg/d Propanolol 160 mg/d Morphine 20 mg/d	ITB 1,100 µg/d	92/81	132/117	0.29/1.43	2	4.5	5	4	2	26
10	f	26	3	Decorticate posturing, opisthotonus, Lateroflexion of head, teeth grinding, Bulldog phenomenon, sweating	255	Baclofen 37.5 mg/d Propanolol 40 mg/d Lorazepam 0.5 mg/d	ITB 200 µg/d	103/98	108/102	0.14/1.29	2	1	4	4	3	100
11	m	23	4	Decorticate posturing, not obeying any comman	417	Baclofen 75 mg/d	ITB 210 µg/d	75/63	99/101	0.57/1.714	3	2	3	4	2.5	39
12	m	16	3	Decorticate posturing, opisthotonus, bulldog phenomenon, sweating	656	Baclofen 100 mg/d Propanolol 40 mg/d Tizanidin 12 mg/d Diazepam 10 mg/d	ITB 1,000 µg/d	87/76	111/118	0/1.43	2	2	4	4	2	213
13	m	22	4	Decerebrate posturing, hypersalivation, teeth grinding	668	Baclofen 87.5 mg/d Propanolol 60 mg/d	ITB 900 µg/d Propanolol 60 mg/d	108/83	122/121	0.29/1.43	3	2	3	4	2	6
14	f	14	3	Left‐sided decorticate posturing, not obeying any commands, loss of pursuit eye movements	130	Baclofen 45 mg/d Alprazolam 0.5 mg/d Tetrazepam 75 mg/d	ITB 170 µg/d Propanolol 20 mg/d Propanolol 40 mg/d Tizanidin 2 mg/d	101/93	111/103	0.14/1.29	3	2	3	3.5	2.5	14
15	m	37	3	Decorticate posturing, sweating bulldog phenomenon	287	Baclofen 50 mg/d Propanolol 20 mg/d	ITB 150 µg/d	84/76	107/102	0.14/1.29	2	1	2	4	1.5	86
16	f	31	3	Decorticate posturing, lateroflexion of head, opisthotonus, not obeying any commands	96	Baclofen 75 mg/d Propanolol 80 mg/d	ITB 250 µg/d Propanolol 10 mg/d	117/82	124/105	0/1.57	3	2	3	4	3	100
17	m	52	3	Decorticate posturing, lateroflexion of head to the right, bulldog phenomenon	99	Baclofen 100 mg/d Buprenorphin 35 µg/hr	ITB 750 µg/d	79//76	116/119	0.43/1.43	2	2	2.5	4	2.5	6
18	m	41	3	Decorticate posturing left‐sided opisthotonus to the left, sweating, bulldog phenomenon	250	Baclofen 100 mg/d Propanolol 100 mg/d Lorazepam 4 mg/d Fentanyl 25 µg/hr	ITB 500 µg/d Fentanyl 25 µg/hr Lorazepam 1 mg/d	89/97	118/111	0.57/1.71	2	1	1	3.5	2.5	5
19	m	17	3	Decorticate posturing, opsithotonus, teeth grinding, not obeying commands	184	Baclofen 100 mg/d Propanolol 120 mg/d	ITB 640 µg/d	75/62	136/124	0/2	3	1	5	3	1	8
20	m	34	4	Decerbrate/decorticate posturing, opisthotonus to the right, teeth grinding, moaning	198	Baclofen 100 mg/d	ITB 370 µg/d	77/82	102/100	0/1.43	2	2	3	4	1.5	4

*Note.* bpm: beats per minute; d: day; f: female; GCS: Glasgow Coma Scale; GOS: Glasgow Outcome Scale; impl.: implantation; ITB: intrathecal baclofen; m: male; No: number; SBP: systolic blood pressure.

a Before/after: before ITB treatment/after ITB pump implantation.

The oral therapeutic schemes of our patients before ITB implantation to treat PSH and at time of discharge from hospital are displayed in Table 1. Oral baclofen was administered to all patients (20/20; 100%) before ITB evaluation and oral propranolol to 15/20 (75%). Median time of complete oral baclofen disposal was 5 ± 3.7 (CI 95% [2.9–7.1], range 0–14) days after implantation of the ITB pump. Median time of complete oral propranolol withdrawal was 24 ± 51.2 (CI 95% [−7.87–55.870], range 5–191) days in 10 of 15 patients; in the remaining 5 of 15 patients, oral propranolol was further administered but with a reduced dosage after discharge from our hospital.

A positive response to ITB, as defined above, via the external pump during the evaluation period could be achieved in 20/20 (100%) patients. Overall, ITB treatment after pump implantation was efficient to control vegetative parameters as well as muscle hypertonia of PSH in all patients. The median percentage of days with tachycardia episodes of ≥100 beats/min could be reduced significantly after ITB pump implantation (5.5% ± 16.4% compared to 24.7% ± 15.6% before ITB treatment; *p* = 0.015). Also, the mean heart rate reduced significantly from 92.8 ± 11.3 to 83 ± 10.3 after implantation of the ITB pump implantation (*p* = 0.04) as well as the mean systolic blood pressure from 118 ± 9.9 to 112.5 ± 8.5 (*p* = 0.008) and the maximum systolic blood pressure from 164.9 ± 18.4 to 151.5 ± 19.7 (*p* = 0.024). The maximum heart rate after ITB treatment did not significantly differ compared to the time before implantation of the ITB pump (130.1 ± 20.7 vs. 138.8 ± 20.9; *p* = 0.218) as did not the median percentage of days with systolic blood pressure >160 mmHg (0.15% ± 2.1% vs. 2.3% ± 4.3%, *p* = 0.109). The number of attacks with motor phenomena per day decreased significantly in all patients (mean score for hypertonic attacks 0.14 ± 0.16 before ITB treatment and 1.42 ± 0.22 after ITB pump implantation; *p* < 0.001).

The time interval between event and first ITB administration via external pump varied between 1 and 80 months (median 5 ± 19.2). The median dosage of ITB during the evaluation phase with the external pump was 144 ± 94.1 (CI 95% [82.236–205.764], range 50–604) µg baclofen/24 hr. On discharge, patients were treated with a median dosage of 280 ± 223 (CI 95% [155.157–404.843], range 145–1,100) µg baclofen/24 hr.

Overall, the median Glasgow Outcome Scale at time of discharge from our hospital was 2 ± 0.5 (CI 95% [1.775–2.225], range 2–3). The median remission phase of the patients at time of admission improved significantly compared to discharge from hospital (1 ± 0.66, CI 95% [0.62–1.38] vs. 3 ± 0.94, CI 95% [2.461–3.539]; *p* = 0.001). Also, the median score of the Ashworth Scale improved significantly from 4 ± 0.340 (CI 95% [3.851–4.149], range 3–4) to 2 ± 0.559 (CI 95% [1.756–2.244], range 1–3; *p* < 0.001) at time of hospital discharge compared to admission.

A follow‐up was available in 18/20 (90%) patients with a median of 46.6 ± 53.242 (CI 95% [23.291–69.959], range 0–213) months after the severe head injury. Two of 20 patients were lost to our follow‐up (treated at a hospital near their home). The median dosage at last follow‐up was 255 ± 258.860 (CI 95% [141.552–368.448], range 25–952.3) µg baclofen/24 hr, the mode of application continuous in 19/20 (95%), and in 1/20 (5%) patient, a bolus application was applied. One patient died during follow‐up resulting from complications due to severity of brain injury. ITB treatment was safe and without any infectious or mechanical complications in 19/20 (95%) patients. In one patient, the intrathecal catheter had to be revised due to dislocation.

## 4 DISCUSSION

We report successful ITB treatment in 20 patients with PSH due to severe traumatic brain injury. After implantation of the ITB pump, oral baclofen could be disposed after a few days, to a lesser extent also oral propranolol which could be withdrawn completely or reduced after a few weeks. Continuous treatment with ITB resulted in an improvement of vegetative parameters and reduction in attacks with motor phenomena as well as functional outcome in all patients. ITB treatment was safe and without major complications in all patients.

Current literature lacks evidence‐based recommendations and guidelines regarding current best practice for therapeutic management of PSH. Perkes and co‐workers reviewed 43 articles referencing the effectiveness of pharmacological management of PSH but stated the poor methodological quality of the studies (Perkes et al., 2010). The best evidence available supported the use of ITB in severely affected patients as orally administered baclofen has its well‐known limitations. Other suggested first‐line oral medications included most opioids, gabapentin, benzodiazepines, centrally acting alpha‐antagonists, and beta‐antagonists. Moreover, according to the literature published so far, one unmet need is the impact of ITB treatment on co‐medication to save other drugs in the management of PSH. We compared oral therapeutic schemas before ITB evaluation and on discharge from hospital in all our patients and analyzed the time and the extent of withdrawal of oral baclofen and oral propranolol as the two most frequently given medication groups. Oral baclofen could be disposed within a few days in 100% of the patients after ITB treatment. Oral propranolol could be completely withdrawn after a few months, starting 3 weeks after ITB treatment on average in about two thirds of the patients. The overlapping administration of ITB and oral propranolol in five patients was useful to manage additional reactive sympathetic phenomena.

During the last years, numerous papers reported on the successful treatment of PSH with ITB in the management of severe brain injury, mainly retrospective case reports (Becker et al., 2000, 1999 ; François et al., 2001; Turner, 2003).

Thereof, only a single study on the efficacy of ITB in controlling PSH was performed prospectively in four patients in the acute recovery phase after traumatic brain injury (Cuny et al., 2001).

The largest study in over 40 patients with severe traumatic brain injury and PSH treated with ITB focussed on predicting factors for long‐term outcome (Turner, 2003). Basically, reported results of this study as well as other small patient series are difficult to compare with our findings due to methodological differences (e.g., the majority of patients being in the acute recovery phase) and different follow‐up periods. Nevertheless overall, vegetative parameters improved in about 92% of reported patient series on effectiveness of ITB on PSH (Becker et al., 2000, 1999 ; Cuny et al., 2001; François et al., 2001; Knutsson et al., 1974). In contrast to case reports published earlier, our patient group consists of 20 patients comprising a mixed patient population regarding the time of referral to our hospital. The majority of patients were referred from a district hospital for a second opinion and evaluation even many years after the event for ITB treatment. Thus, the time interval between event and first ITB administration via external pump varied considerably in our patient group (range 1–80 months).

Regarding the impact of ITB on spasticity, the Ashworth Scale on admission compared to discharge from hospital in our patient group was roughly comparable to results of earlier studies in smaller patient series with a decrease of 2 points on average (Becker et al., 1997; Turner, 2003). Our results with an improvement of the Glasgow Outcome Scale in patients referred even after months or years of the initiating might be an argument that ITB treatment seems still to be a successful therapeutic option in these patients.

This study contains several limitations: The data, also efficacy of ITB treatment, were acquired retrospectively. In addition, the results are limited to our single rehabilitation center and comprise a heterogenous patient population regarding the time of initiating the ITB treatment. However, despite the long time period of 18 years and the heterogeneity of the population, this is the first study evaluating the impact of ITB treatment on cutting back or dispensing with other drugs in order to optimize the management of PSH and possibly improve functional outcome. Administration and treatment with ITB were effective and safe in controlling PSH in all our patients, unless they were in the early recovery or persistent vegetative state, and no complications or adverse events occurred.

Although ITB treatment should be already initiated in the acute recovery phase, our data might indicate that ITB might also be a successful therapeutic option to control PSH even years after the initiating event and possibly still improve functional outcome. Moreover, our results underline the necessity of systematic evaluation of treatment regimes to optimize the management and outcome of patients with PSH. Further work with prospective multicenter and large sample studies is needed to assess the efficacy of therapies and to elucidate the putative role of ITB in the management of PSH. Subsequently, a consensus approach to develop expert‐based management guidelines and recommendations should be pursued to optimize pharmacotherapy, rehabilitation interventions, and outcome.

## CONFLICT OF INTERESTS

All authors declare no conflict of interests regarding this article.
